# A Case of Salt-Wasting 21-Hydroxylase Deficiency With Resistance to Aldosterone due to Urinary Tract Infection

**DOI:** 10.7759/cureus.11763

**Published:** 2020-11-29

**Authors:** Urara Shimakawa, Keiichi Shigehara, Yasuhiro Kawabe, Kazutaka Ouchi, Jun Mori

**Affiliations:** 1 Department of Pediatrics, Ayabe City Hospital, Ayabe, JPN; 2 Department of Pediatrics, Kyoto Prefectural University of Medicine, Kyoto, JPN

**Keywords:** 21-hydroxylase deficiency, hyperkalemia, fludrocortisone, urinary tract infection, pseudohypoaldosteronism

## Abstract

Classic salt-wasting 21-hydroxylase deficiency (21-OHD) often requires fludrocortisone (FC) replacement. However, the optimal dose of FC varies between patients and the dose needs to be adjusted depending on the degree of symptoms. Further, the aldosterone resistance due to urinary tract infections causes salt-wasting symptoms. We recently encountered a patient with 21-OHD who required up to 0.36 mg/day of FC in order to control hyperkalemia despite adequate hydrocortisone (HC) administration. This condition was presumed to be due to aldosterone resistance complications associated with urinary tract infections. Thus, if the initial treatment of 21-OHD with HC and FC is resistant, then one should consider complications that may cause aldosterone resistance, such as urinary tract infections.

## Introduction

21-hydroxylase deficiency (21-OHD) is the most common cause of congenital adrenal hyperplasia (CAH). The enzyme 21-hydroxylase is involved in the synthesis of cortisol and aldosterone in the adrenal glands. A patient with 21-OHD cannot synthesize cortisol and aldosterone, and therefore exhibits vomiting, diarrhea, and salt-wasting symptoms (dehydration, hyponatremia, and hyperkalemia) during the neonatal period [1]. Cases exhibiting salt-wasting symptoms require fludrocortisone (FC) replacement [2]. Further, aldosterone resistance due to renal anomalies and/or urinary tract infections (UTI) can cause salt-wasting symptoms and this is called secondary pseudohypoaldosteronism (PHA) [3], and these conditions can occur together. In such cases, this may mask the diagnosis of 21-OHD and make the initial treatment of 21-OHD difficult [4]. Herein, we describe a case of a patient with 21-OHD complicated with UTI who required up to 0.36 mg/day dose of FC in order to control hyperkalemia despite adequate hydrocortisone (HC) administration.

## Case presentation

The patient was a 10-day-old boy with no known family history. He was delivered vaginally at another facility on the fourth day of gestational week 39. His birth weight was 2734 g and he had an Apgar score of 8/9. At 10 days of age, the patient was seen by a physician at the same facility for his two-week checkup, during which he was found to be not gaining weight at a healthy rate (+9 g/day). On the same day, a newborn mass screening test found that he may have 21-OHD. The patient was then taken to our hospital’s emergency department.

**Figure 1:**
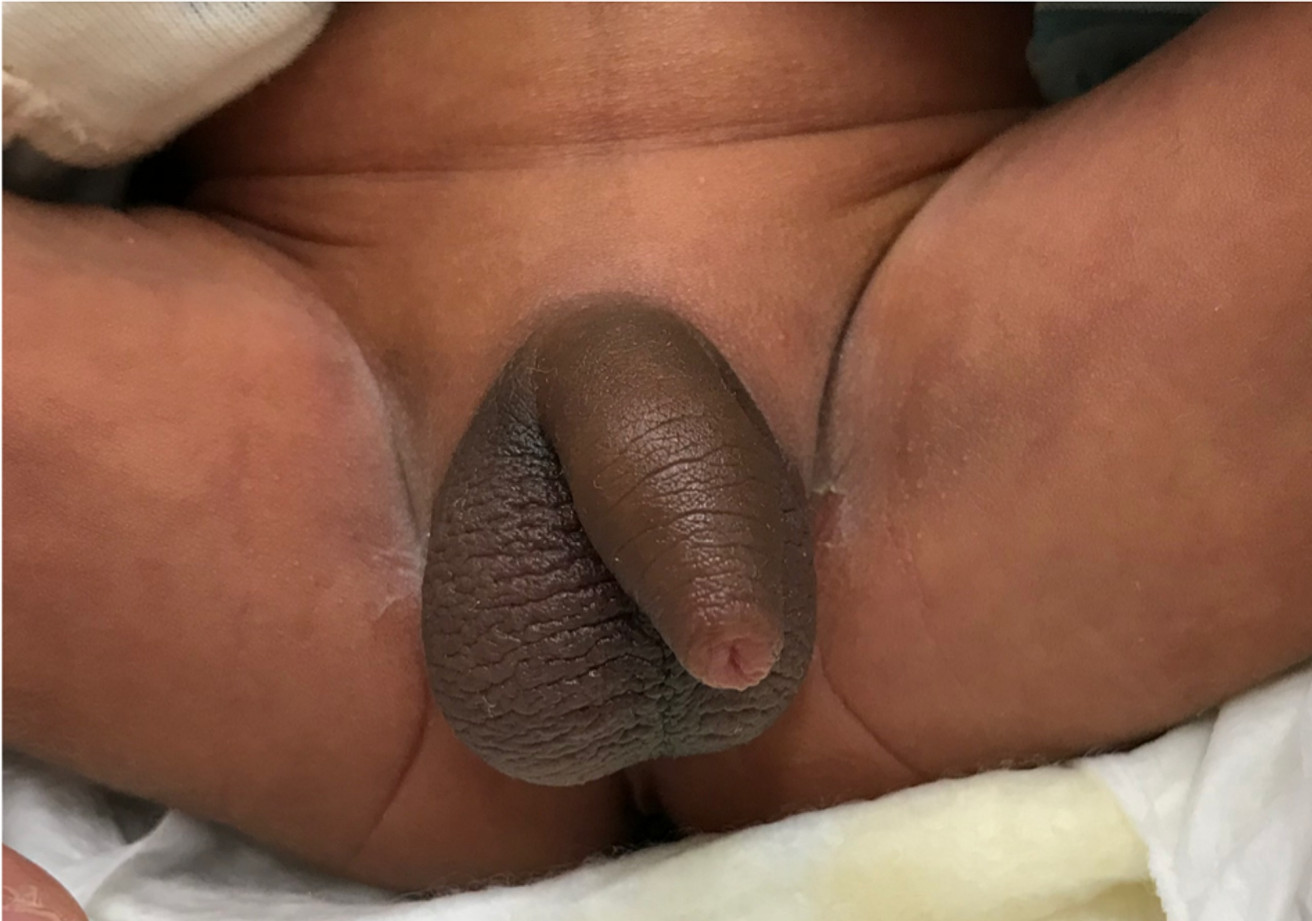
The photograph of pigmentation of the genital area show

The physical findings at admission were as follows: body weight, 2634 g; body temperature, 38.2 °C; heart rate, 147 bpm; blood pressure, 74/45 mmHg; respiratory rate, 40 breaths per minute; SpO_2_, 100% (room air); sunken anterior fontanelle; no conjunctival congestion; mild conjunctival jaundice; and no pharyngeal redness. Regarding the chest, there were regular heart sounds with no murmurs and clear breath sounds. The abdomen was soft and flat with normal peristaltic sounds. Pigmentation of the genital area was observed (Figure 1).

Peripheral body areas were not cold to the touch. Dry skin was present.

Laboratory tests were performed after admission. A mass screening test performed on day four found a very high concentration of 17-hydroxyprogesterone (17-OHP; direct method) of 279.5 ng/mL. A markedly low sodium level (125 mEq/L) and markedly high potassium level (8.5 mEq/L) were also observed. At a later date, endocrine testing results showed that the levels of the following items were elevated: testosterone, 514.8 ng/dL; aldosterone, 103.9 ng/dL; adrenocorticotropic hormone (ACTH), 323.0 pg/mL; and renin, >45 ng/(mL·h). The levels of the following parameters were within the normal ranges: cortisol, 14.9 μg/dL; and estradiol, 28.4 pg/mL. Additionally, urinary steroid profile using gas chromatography-mass spectrometry showed that urinary 3α, 17α, 20α-pregnanetriolone (Ptl) (21.818 mg/g Cre), pregnanediol (PD5) (0.230 mg/g Cre) and 11β-hydroxyandorosterone (11-OHAn) (4.894 mg/g Cre) (Table 1).

**Table 1:** Laboratory findings WBC, white blood cell; Hb, hemoglobin; Hct, hematocrit; Plt, platelets; BE, base excess; Lac, lactate; LDH, lactate dehydorogenase; Alb, albumin; BUN, blood urea nitrogen; Cre, creatinine; Na, sodium; K, potassium; Cl, chloride; Ca, calcium; T-Bil, total billirubins; AST, aspartate aminotransferase; ALT, alanine aminotransferase; ALP, alkaline phosphatase; ACTH, adrenocorticotropic hormone; 17-hydroxyprogesterone; Ptl, 3α, 17α, 20α-pregnanetriolone; 11-OHAn, 11β-hydroxyandorosterone Normal ranges are shown in parentheses.

Hematology			Biochemistry			Endocrine			Normal range
WBC	10,710	/μL	LDH	428	IU/L	estradiol	28.4	pg/mL	(<62.4)
Hb	18	g/dL	Alb	4.7	g/dL	testosterone	514.8	ng/dL	(0.12-0.21)
Hct	50.9	%	BUN	18.6	mg/dL	cortisol	14.9	μg/dl	(2-15)
Plt	55.5	×10^4^/μL	Cre	0.48	mg/dL	aldosterone	103.9	ng/dL	(3.0-15.9)
			Na	125	mEq/L	renin	>45	ng/(mL·h)	(0.3-2.9)
			K	8.5	mEq/L	ACTH	323	pg/mL	(12.6-35)
Venous blood gas			Cl	95	mEq/L	17-OHP	279.3	ng/ml	(0.6-2.8)
pH	7.353		Ca	11.3	mg/dL				
pCO_2_	50.1	mmHg	T-Bil	16.14	mg/dL	urinary steroid profile			
HCO_3_	27.9	mmol/L	AST	35	IU/L	Ptl	21.818	mg/g Cre	(0.000-0.003)
BE	1.1	mmol/L	ALT	12	IU/L	11-OHAn	4.894	mg/g Cre	(0.009-0.120)
Lac	2.6	mmol/L	ALP	1490	IU/L				

**Figure 2:**
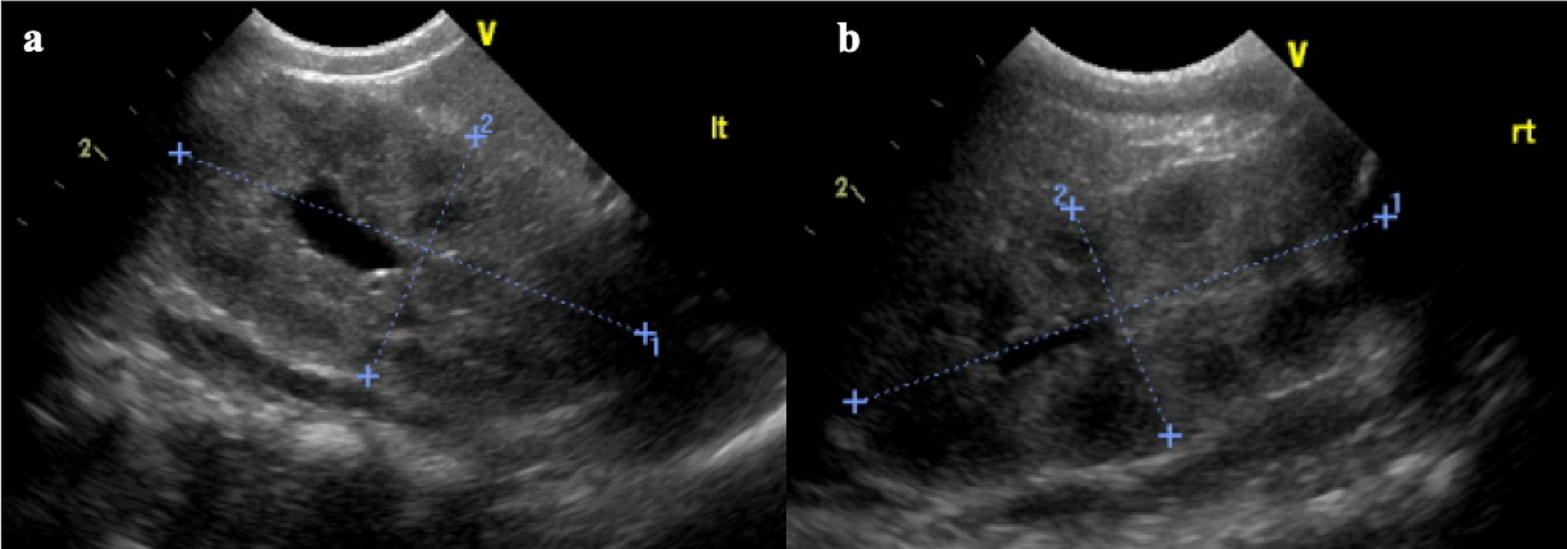
Ultrasound imaging of hydronephrosis (a) Left kidney has renal pelvis dilation, showing grade 1 hydronephrosis. (b) Right kidney without renal pelvis dilation.

At the beginning of hospitalization, based on the results of mass screening testing and clinical symptoms, the patient was diagnosed with classic 21-OHD. The patient was considered to have acute adrenal failure due to hyperkalemia and hyponatremia. Intravenous HC was administered rapidly at 100 mg/m^2^. Thereafter, a 100 mg/(m^2^·day) dose of HC and saline were administered by continuous infusion. Glucose-insulin therapy was initiated to treat the hyperkalemia. On Hospitalization Day 3, his body temperature reached 39.2 °C. His urinalysis revealed leukocytes and bacteriuria (*Streptococcus agalactiae* 100,000 CFU/mL collected by catheterization), and renal ultrasonography found grade 1 hydronephrosis of the left kidney (Figure 2).

**Figure 3:**
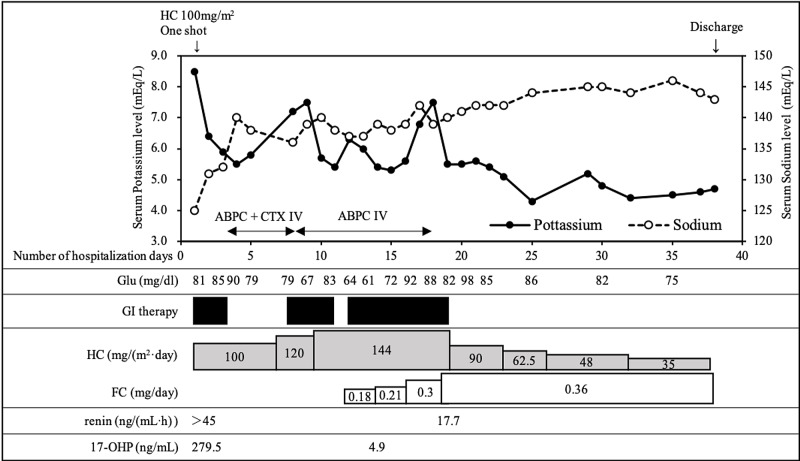
Post-admission course ABPC, ampicillin; CTX, cefotaxime; Glu, blood glucose level; GI, glucose-insulin; HDC, hydrocortisone; FC, fludrocortisone; 17-OHP, 17-hydroxyprogesterone

As sepsis workup, we also performed bacterial cultures in blood and spinal fluid; no bacteria were detected in these samples. Therefore, an intravenous antibiotics treatment for UTI was started. The patient was able to keep his body temperature below 37.5℃ on Hospitalization Day 5, but the hyperkalemia persisted. At that time, we considered pseudohyperkalemia by blood sampling, acute renal failure, urinary obstruction, hemolysis, and drug-induced as the differential diagnosis of hyperkalemia, but all of them were denied. The doses of HC and FC are dependent on the individual patient and managed based on the severity of clinical symptoms [5]. Consequently, using serum potassium levels as an indicator, the dose of HC was increased to 120 mg/(m^2^·day) on Hospitalization Day 7 and then further increased to 144 mg/(m^2^·day) on Hospitalization Day 9. Despite these dose increases, serum potassium levels could not be controlled; therefore, oral administration of 0.18 mg/day FC and 0.1 g/(kg·day) sodium chloride was initiated. On Hospitalization Day 18, the serum potassium level was 7.5 mEq/L, and the transtubular potassium gradient (TTKG) and fractional excretion of sodium were 2.5 and 9.29 %, respectively. Since these findings suggested low aldosterone bio-activity, the dose of FC was increased to 0.36 mg/day. Thereafter, serum potassium levels gradually decreased. In addition, a mass screening test was again performed again on Hospitalization Day 14, revealing that the 17-OHP concentration (direct method) decreased to 4.9 ng/mL. On Hospitalization Day 18, the serum renin activity had reduced to 17.7 ng/(mL·h). Thereafter, the dose of HC was gradually reduced to 35 mg/(m^2^·day). The patient was discharged on Hospitalization Day 38 (Figure 3).

Following discharge, the doses of HC and FC were reduced to maintenance doses of 12 mg/(m^2^·day) and 0.2 mg/day, respectively. The administration of sodium chloride was discontinued. The patient has received regular follow-up care, but no notable findings and no electrolyte abnormalities have been observed to date, and the low TTKG improved to 7.4.

## Discussion

When we see patients with high levels of 17-OHP, in addition to 21-OHD, we should distinguish P450 oxidoreductase deficiency (PORD), transient hyper-17-hydroxyprogesteronemia, 3β-hydroxysteroid dehydrogenase (3β-HSD) deficiency, and 11β-hydroxylase (11β-OHD) deficiency. For 21-OHD, PORD and transient hyper-17-hydroxyprogesteronemia, differential diagnosis procedures have been reported using urinary profile, specifically, Ptl level and 11-OHAn/PD5 ratio [6]. In this case, Ptl and 11-OHAn/PD5 ratio were significantly elevated, a finding typical of 21-OHD. In patients with PORD, electrolytes are normal and neonatal adrenal insufficiency is rare. The 46, XY karyotype in boys with PORD has been reported to be associated with early craniosynostosis, characteristic facies, humeral-radial synostosis, and joint contracture [7]. Our case did not have any of these characteristic findings of PORD. In contrast to 21-OHD, 3β-HSD deficiency causes incomplete virilization, for example genital abnormalities, in boys with 46XY karyotype [8]. 11β-OHD is endocrinologically differentiated for 21-OHD by decreased plasma renin activity and aldosterone levels [9]. In urinary steroid profile analysis based on several test results until three to four months after birth, continued elevated 17α-hydroxypregnenolone (17-OHP5) and tetrahydro-11-deoxycorticosterone (THDOC) is also useful for diagnosis of 3β-HSD deficiency and 11β-OHD, respectively [5]. Both 17-OHP5 and THDOC in urinary steroid profile were low in our case. We made a final diagnosis of 21-OHD from these findings, however, as genetic diagnosis is useful for differentiation of PORD, 3β -HSD deficiency and 11β-OHD [10], we should consider performing genetic testing.

In general, serum aldosterone and serum cortisol levels are expected to be low in patients with CAH and elevated 17-OHP and ACTH levels are important for diagnosis. On the other hand, in patients with PHA, serum aldosterone levels are significantly elevated, and 17-OHP and serum cortisol levels are expected to be normal. However, previous reports indicate that patients with CAH sometimes have normal to very high levels of aldosterone and normal levels of cortisol, and this may make the diagnosis and initial treatment of CAH difficult [4]. Similarly, the laboratory tests of our patient revealed elevated serum aldosterone and normal serum cortisol levels. There are two possible explanations for elevated serum aldosterone levels in this patient. Firstly, the high levels of aldosterone in patients with CAH can be seen if there is co-existing PHA that caused UTI. Secondly, it has been recently reported that there are possible elements of cross-reactivity between different steroid precursors that cause interference in the aldosterone assay [11]. Additionally, the normal level of serum cortisol of our patient can be explained by the high level of 17-OHP. 17-OHP can produce the elevation of 21-deoxycortisol, which shows clinically relevant cross-reactivity for cortisol in patients with 21-OHD [12].

In patients with classic salt-wasting 21-OHD, if a high-dose administration of HC does not improve the electrolyte imbalance in the acute-stage treatment, then an administration of FC is required [10]. However, the dose needs to be adjusted depending upon the severity of the symptoms, and the optimal dose varies because sensitivity to FC varies greatly between individuals [13]. The diagnosis/treatment guidelines for 21-OHD by the Mass Screening Committee of the Japanese Society for Pediatric Endocrinology recommend administering 0.025-0.2 mg/day of FC [5]. Our literature search found a patient who required a maximum dose of 0.35 mg/day [14]. In the present case, 0.3 mg/day of FC did not improve hyperkalemia; hence, the dose needed to be increased up to 0.36 mg/day. The reason was speculated as follows: the expression of aldosterone receptors in the distal nephron is limited in a newborn and neonatal nephrons are known to be resistant to the actions of aldosterone [15]. Therefore, the required dose of FC is higher, especially at 0 to six months, than the dose administered after that; moreover, the dose range is wide because it depends on the large individual differences in sensitivity to aldosterone [14]. In addition, this resistance to aldosterone may be exacerbated by the presence of PHA caused by UTI and/or urinary tract abnormalities [16]. In our case, the combination of 21-OHD induced mineralocorticoid deficiency and UTI induced aldosterone resistance made it difficult to control hyperkalemia. For treating infants with hyperkalemia, TTKG is a useful indicator of renal aldosterone bio-activity, and a TTKG value less than 4.9 in infants indicates a low aldosterone bio-activity [17]. It has also been reported that TTKG increases above 4 within 4 h and in most cases within 2 h after administration of physiological doses of mineralocorticoids to patients with adrenal insufficiency [18]. Our case showed the presence of aldosterone resistance, as the serum potassium level and TTKG were 7.5 mEq/L and 2.5, respectively, despite the administration of 144 mg/(m2·day) HC and 0.3 mg/day FC. In a retrospective measurement of 17-OHP (direct method) at that time, we found that the 17-OHP level was reduced to 4.9 ng/mL. It has been previously reported that serum 17-OHP is the optimal indicator of effective glucocorticoid activity [19]. Thus, a sufficient dose of HC was administered in the present case.

Incidentally, it has been found that the expression of mineralocorticoid receptors gradually increases and plateaus at approximately one year of age [15]. For this reason, certain adult patients no longer require FC even if they did as a pediatric patient. It should be noted that studies have found that FC overdose can lead to an onset of hypertension and myocardial hypertrophy [20]. Thus, the optimal dose of FC needs to be adjusted depending on parameters such as serum electrolyte levels, plasma renin levels, weight gain, edema, and blood pressure. In our case, during hospitalization, the patient’s blood pressure persisted at 100-120/60-80 mmHg, but easily returned to normal as the FC dose was reduced. On Hospitalization Day 29, an echocardiography was performed; however, myocardial wall thickening was not observed. The patient has since gone on to live without any side effects that suggest an overdose of FC.

## Conclusions

In conclusion, the patient with classic salt-wasting 21-OHD and UTI required 0.36 mg/day dose of FC in the initial treatment. If it is difficult to control the electrolyte levels in a patient with 21-OHD, despite administration of high-dose HC and adequate doses of FC, one should consider that the patient may have complications of aldosterone resistance, such as a UTI.
